# Fluorescence Enhancement of Fluorescent Unnatural Streptavidin by Binding of a Biotin Analogue with Spacer Tail and Its Application to Biotin Sensing

**DOI:** 10.1155/2014/165369

**Published:** 2014-03-20

**Authors:** Xianwei Zhu, Hiroaki Shinohara

**Affiliations:** ^1^Graduate School of Innovative Life Science for Education, University of Toyama, 3190 Gofuku, Toyama 930-8555, Japan; ^2^Graduate School of Science and Engineering for Research, University of Toyama, 3190 Gofuku, Toyama 930-8555, Japan

## Abstract

We designed a novel molecular biosensing system for the detection of biotin, an important vitamin by the combination of fluorescent unnatural streptavidin with a commercialized biotin-(AC_5_)_2_-hydrazide. A fluorescent unnatural amino acid, BODIPY-FL-aminophenylalanine (BFLAF), was position-specifically incorporated into Trp120 of streptavidin by four-base codon method. Fluorescence of the Trp120BFLAF mutant streptavidin was enhanced by the addition of biotin-(AC_5_)_2_-hydrazide with the concentration dependent, whereas fluorescence enhancement was not observed at all by the addition of natural biotin. It was considered that the spacer tail of biotin-(AC_5_)_2_-hydrazide may disturb the fluorescence quenching of the Trp120BFLAF by Trp79 and Trp108 of the neighbor subunit. Therefore, biotin sensing was carried out by the competitive binding reaction of biotin-(AC_5_)_2_-hydrazide and natural biotin to the fluorescent mutant streptavidin. The fluorescence intensity decreased by increasing free biotin concentration. The result suggested that molecular biosensor for small ligand could be successfully designed by the pair of fluorescent mutant binding protein and ligand analogue.

## 1. Introduction

Biotin is a water-soluble B-complex vitamin, also known as Vitamin B-7 or Vitamin H. It not only is an essential component for synthesis of Vitamin C but also works as a coenzyme in the synthesis of fatty acids, isoleucine, and valine and plays an important role in gluconeogenesis. Biotin deficiency induces alopecia, conjunctivitis, dermatitis, depression, lethargy, hallucination, numbness, and tingling of the extremities. Biotin stays in the human body for 3~6 hours because of its water solubility [1–5]. Streptavidin is a tetrameric biotin binding protein isolated from* Streptomyces avidinii.* The dissociation constant (*K*_*d*_) of streptavidin to biotin is on the order of 10^−15^ mol/L that is extraordinarily high affinity. Then streptavidin-biotin binding reaction has been widely applied to biotin detection, biomolecule purification, protein assays, diagnostics, and drug delivery by coupling with various reporter probes, such as fluorescence dyes, radioactive elements, or enzymes [6–8]. Therefore, more rapid convenient methods for the determination of biotin are further required.

**Figure 1:**
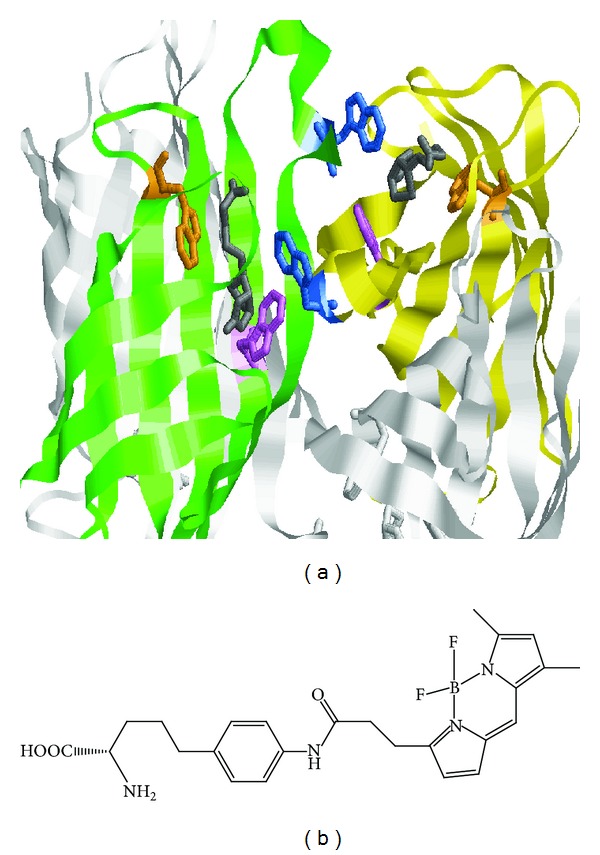
(a) The homo tetrameric constitution of a natural streptavidin (PDB ID: 1SWE). Subunit A was colored green, subunit B was colored yellow; to be easy to see, both subunits C and D were colored white. Biotin was colored with gray. On the other hand, Trp79, Trp108, and Trp120 in subunits A and B were colored orange, violet, and blue, respectively. Obviously the Trp120 was close to the Trp79 and the Trp108 of the neighbor subunit. (The distance from Trp120 to Trp79 of the neighbor subunit is 8.74 Å and Trp120 to Trp108 of the neighbor subunit is 6.78 Å.) (b) The chemical structure of the BFLAF.

The incorporation of functional unnatural amino acids into proteins is a powerful and versatile technique for designing a functional protein for biosensor and protein structural and functional analysis. A number of researchers have synthesized proteins containing unnatural amino acids at desired positions by using an amber suppression technique [9–14]. Alternatively, four-base codon method, an excellent method, can perform the site-directed introduction of single or multiple functional unnatural amino acids that possessed fluorescent, oxidation-reduction to provide a new function into the protein. In this method, one or multiple four-base codons can be introduced into an assigned position of a protein gene. The full-length protein containing the unnatural amino acid could be produced when the four-base codon is successfully decoded by the unnatural aminoacyl-tRNA having the corresponding four-base anticodon. On the other hand, when the first three bases of the four-base codon are decoded as a three-base codon by a cognate naturally occurring aminoacyl-tRNA, a frame shift occurs that causes the emergence of a stop codon resulting in the termination of peptide elongation. Therefore, the full-length protein could be obtained only when the four bases are successfully decoded as a single codon. The four-base codon method is advantageous over the amber codon suppression technique because unnatural amino acids can be introduced with higher efficiencies. Moreover, using orthogonal four-base codons, even more than three unnatural amino acids can be introduced into single proteins. Our collaborators Hohsaka et al. have reported the synthesis of fluorescent mutant streptavidin by introducing a fluorescent unnatural amino acid named BODIPY-FL-aminophenylalanine (BFLAF) that BODIPY-FL bound to aminophenylalanine into streptavidin with four-base codon method (Figure 1(b)) [15–22].

On the other hand, it was recently reported that tryptophan could quench the fluorescence from the fluorophore which was modified to the target protein at the position near the tryptophan [23].

In this study, the BFLAF was incorporated into streptavidin at the Trp120 position with the four-base codon method. The synthesized and purified Trp120BFLAF mutant streptavidin showed fluorescence certainly as we expected. And the fluorescent mutant streptavidin had enough biotin binding activity as about 70% as a wild type of streptavidin. We further discovered that fluorescence of the Trp120BFLAF mutant streptavidin was enhanced when the biotin analogue with a (AC_5_)_2_-hydrazide tail was bound to the fluorescent binding protein, whereas natural biotin binding did not induce the fluorescence intensity change. We then speculate that the fluorescence quenching of Trp120BFLAF by Trp79 and Trp108 of the neighbor subunit may be disturbed by the long spacer tail of the biotin analogue (Figure 1(a)). Finally, we applied this fluorescence enhancement of the Trp120BFLAF mutant streptavidin by binding biotin-(AC_5_)_2_-hydrazide to the molecular biosensing system for free biotin based on the competitive binding reaction of free biotin and biotin-(AC_5_)_2_-hydrazide. Sensitive detection of free biotin was performed in the concentration range of 20~100 nM.

## 2. Materials and Method

### 2.1. Materials

The DNAs of wild type and Tyr83 and Trp120 mutant streptavidin and DNA of the tRNA having four-base anticodon and BODIPY-FL-aminophenylalanyl-pdCpA were obtained from laboratory of Professor Hohsaka of Japan Advanced Institute of Science and Technology (JAIST). Alkaline phosphatase-labeled anti-mouse IgG and Magne His Ni-particles were obtained from Promega. Polymerase chain reaction (PCR) enzyme (Vent DNA polymerase) and T7 RNA polymerase were purchased from New England Biolabs. T4 RNA ligase was from Takara Bio. Anti-T7-tag antibody was obtained from Novagen. Biotin-(AC_5_)_2_-hydrazide was purchased from DOJINDO.

### 2.2. Preparation of Mutant Streptavidin

The mRNAs of streptavidin containing four-base codon at Tyr83 and Trp120 were synthesized using their DNAs by T7 RNA polymerase. The tRNA was derived from yeast phenylalanine that contained the corresponding four-base anticodon and lacked 3′-terminal dinucleotide was also synthesized using its DNA by T7 RNA polymerase; thereafter, the BODIPY-FL-aminophenylalanyl-pdCpA was bound to the tRNA by T4 RNA ligase for obtaining BODIPY-FL-aminophenylalanyl-tRNA. A cell-free translation was carried out by adding streptavidin mRNA and BODIPY-FL-aminophenylalanyl-tRNA into the reaction mixture containing* E. coli *extract. The reaction mixture (10 *μ*L) contained 7 *μ*L of* E. coli *extract, 0.1 mM each of naturally occurring amino acids, 15 pmol of mRNA, and 0.2 nmol of BODIPY-FL-Aminophenylalanyl-tRNA. After incubation for 60 min at 37°C, the product was purified by MagneHis Ni-Particles, MicroSpin G25 column, and analyzed by western blotting using the anti-T7-tag antibody and an alkaline phosphatase-labeled secondary antibody.

### 2.3. Confirmation of the Biotin Binding Activity of the Fluorescent Mutant Streptavidin

The biotin binding activity of the wild type and mutant streptavidins was confirmed with dot blot analysis. Two times the amount of the mutant streptavidins as compared with wild type streptavidin were immobilized on the cellulose nitrate to bind with alkaline phosphatase-labeled biotin and then assayed with western blue for coloring. The coloring image of the dot on the blotting membrane was taken with a scanner and analyzed the spots density by the commercialized imaging software, the NIH ImageJ software.

### 2.4. Fluorescence Analysis of the Mutant Streptavidins

The biotin-(AC_5_)_2_-hydrazide was first dissolved in DMSO. 10 *μ*L of the mutant streptavidin solution was mixed with 30 *μ*L of the biotin-(AC_5_)_2_-hydrazide solution and 10 *μ*L of the biotin solution and then diluted to 100 *μ*L with HKM buffer (100 mM KCl, 25 mM Hepes, 5 mM MgCl_2_, pH adjusted to 7.4 with KOH). A fluorescence spectrometer (JASCO FP-6500) was used to measure excitation and emission spectra of the sample solutions with the slit width in 5 nm for both excitation and emission.

## 3. Results and Discussion

### 3.1. Confirmation of the Mutant Streptavidin Productions

**Figure 2:**
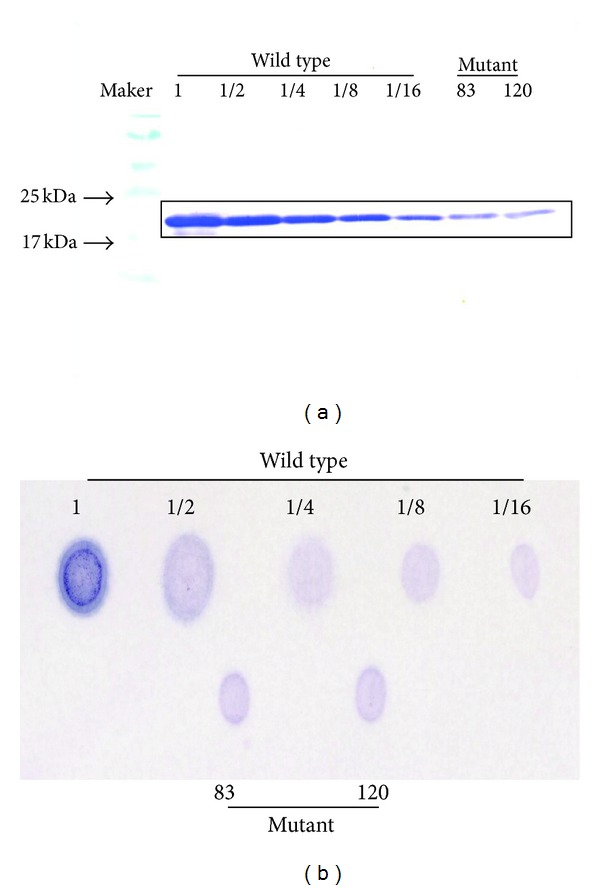
(a) The result of western blotting analysis for wild type, Tyr83BFLAF and Trp120BFLAF mutant streptavidins. BFLAF was position-specifically incorporated into streptavidin by using a CGGG four-base codon. The bands of the wild and mutant streptavidins were observed around 20 kDa. (b) The result of dot blotting. The biotin binding activities of these fluorescent mutant streptavidin were evaluated by a dot blot analysis using an alkaline phosphatase-labeled biotin. The protein amounts of Tyr83BFLAF and Trp120BFLAF mutant streptavidins blotted on the membrane were 15.2% and 14.4% of that of wild type streptavidin.

The wild type and Tyr83BFLAF and Trp120BFLAF mutant streptavidins were synthesized by* in vitro* translation and purified by using the His-tag affinity nickel particles. The synthesized mutant streptavidins were then subjected to SDS-PAGE to compare the molecular weight. The bands of two mutant streptavidins were confirmed by western blotting, in which the T7-tag of the N-terminal of the mutant streptavidin was recognized with the anti-T7-tag antibody from mouse and the secondary antibody for mouse antibody that was labeled with alkaline phosphatase. The full length of streptavidin (20 kDa) was obtained only in the presence of BFLAF aminoacyl-tRNA in the translation system (Figure 2(a)). The western blotting data indicated that the BFLAF was incorporated into the mutant streptavidins at each specific position to make the full length of streptavidin. The bands of the wild type and two mutant streptavidins in blotting membrane were seen at the almost same molecular weight of 20 kDa. The incorporation efficiency of the BFLAF, however, depended on the incorporated site. The relative yields of the full-length streptavidins obtained in the presence of the BFLAF aminoacyl-tRNA were determined by comparing the band density of the full-length products as compared with those of serially diluted wild type streptavidin. The protein yields of Tyr83BFLAF and Trp120BFLAF mutants were were analyzed to 7.6% and 7.2%, respectively, as compared with the band density of wild type streptavidin as 100% with NIH ImageJ software.

The biotin binding activities of the wild type and the mutant streptavidins were evaluated by dot blotting using alkaline phosphatase-labeled biotin. For confirmation of the biotin binding activities of the mutant streptavidins accurately and easily, 2 times the amount of each mutant streptavidin solution as compared with wild type streptavidin solution were blotted on the membrane. Therefore, the protein amounts of Tyr83BFLAF and Trp120BFLAF mutant streptavidins blotted on the membrane were 15.2% and 14.4% of that of wild type streptavidin from western blotting analysis. The blot density of Tyr83BFLAF and Trp120BFLAF was 9.6% and 9.8%, respectively, as compared with the wild type streptavidin with NIH ImageJ software. Therefore, the biotin affinities of Tyr83BFLAF and Trp120BFLAF mutant streptavidins were evaluated to 63% and 68%, respectively, as compared with the wild type streptavidin. The results indicated that the streptavidin mutants containing BFLAF at positions of Tyr83 and Trp120 retained the biotin binding activity as high as wild type streptavidin (Figure 2(b)). These positions locate at surface regions of the beta-barrel structure or at an interface region of a biotin binding pocket that may be little influential in biotin binding activities.

### 3.2. Results of Fluorescence Analysis of Mutant Streptavidins

**Figure 3:**
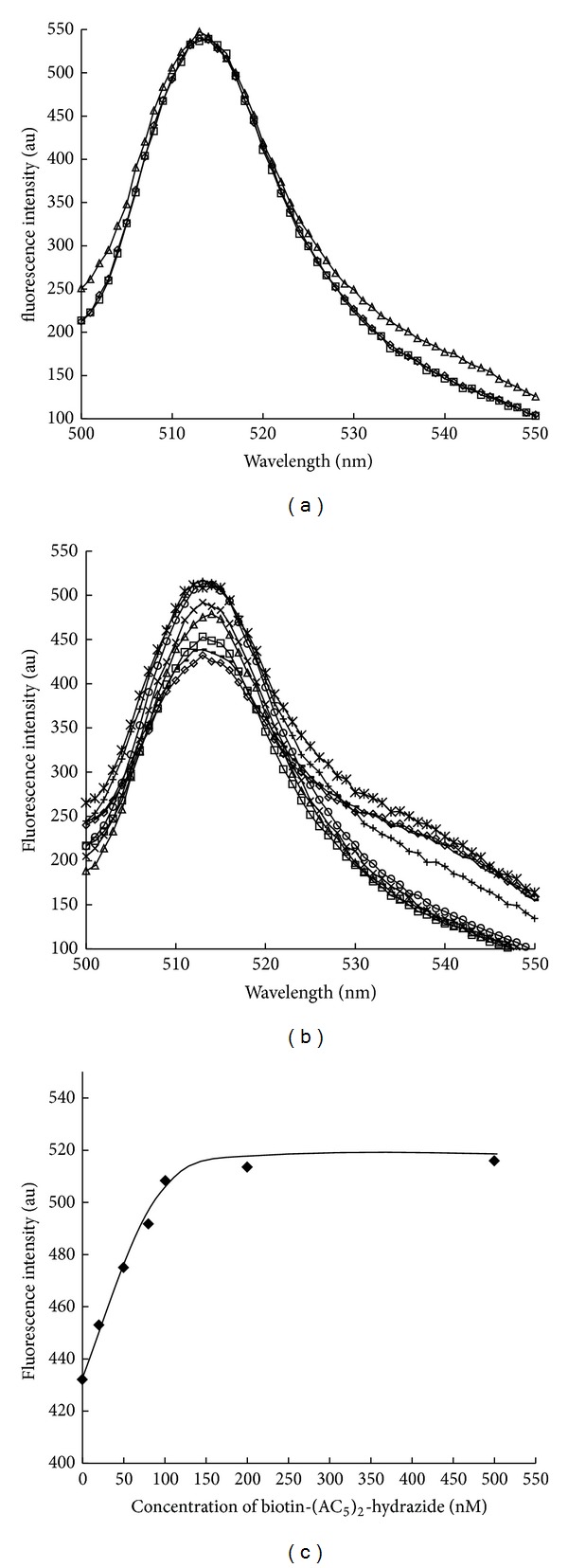
(a) Fluorescence spectrum of the Tyr83BFLAF mutant streptavidin only (–◯–); fluorescence spectrum of the Tyr83BFLAF mutant streptavidin in the presence of 100 nM of natural biotin (–▵–) or 100 nM of biotin-(AC_5_)_2_-hydrazide (–□–); there was no change in the fluorescence intensity (au) upon addition of natural biotin or biotin-(AC_5_)_2_-hydrazide for Tyr83BFLAF mutant streptavidin. (b) Fluorescence spectra of the Trp120BFLAF mutant streptavidin only (–*◊*–), in the presence of 100 nM of biotin (–■–), 20 nM (–□–), 50 nM (–▵–), 80 nM (–×–), 100 nM (–∗–), 200 nM (–◯–), or 500 nM (–+–) of biotin-(AC_5_)_2_-hydrazide. Marked fluorescence enhancement was observed for the Trp120BFLAF mutant streptavidin by addition of biotin-(AC_5_)_2_-hydrazide. (c) Dependence of the fluorescence enhancement of Trp120BFLAF mutant streptavidin on the concentration of added biotin-(AC_5_)_2_-hydrazide (20 nM to 100 nM) was observed. The fluorescence intensities of the Trp120BFLAF mutant streptavidins in the presence of various concentration of biotin-(AC_5_)_2_-hydrazide at 514 nm were plotted. All of the fluorescence spectra of mutant streptavidins were excited at 490 nm.

In this study, the biotin-(AC_5_)_2_-hydrazide was used as a biotin analogue with a long spacer tail in which the hydrazide group was inactive to protein, and the double of 6-aminohexanoic acid linker was expected to disturb the fluorescence quenching of the Trp120BFLAF by Trp79 or Trp108 of the neighbor subunit. Tyr83BFLAF was used as a contrast. Before the experiment of fluorescence enhancement of the fluorescent mutant streptavidin by the addition of biotin-(AC_5_)_2_-hydrazide, 100 nM of natural biotin was added to the solution of mutant streptavidins for confirming no effect of natural biotin. Actually, the effect of fluorescence intensity from natural biotin was little (Figures 3(a) and 3(b)).

The Fluorescence spectra of fluorescent mutant streptavidins (Tyr83BFLAF and Trp120BFLAF) upon the addition of biotin-(AC_5_)_2_-hydrazide were next measured. There was almost no change in the fluorescence intensity of Tyr83BFLAF mutant streptavidin in the presence (100 nM) and absence of biotin-(AC_5_)_2_-hydrazide (Figure 3(a)).

In contrast, the Trp120BFLAF mutant streptavidin showed the marked fluorescence enhancement upon the addition of biotin-(AC_5_)_2_-hydrazide (100 nM). The fluorescence intensity of the Trp120BFLAF mutant streptavidin was 1.18 times higher than that in the absence of biotin-(AC_5_)_2_-hydrazide (100 nM) (Figure 3(b)). The fluorescence enhancement of Trp120BFLAF mutant streptavidin was observed upon the addition of 20 nM biotin-(AC_5_)_2_-hydrazide. Concentration dependence for fluorescence enhancement of Trp120BFLAF mutant streptavidin upon the addition of biotin-(AC_5_)_2_-hydrazide was shown in Figure 3(c). Though this fluorescence enhancement of the Trp120BFLAF mutant streptavidin may be due to the disturbance of the fluorescence quenching of Trp120BFLAF by Trp79 or Trp108 of the neighbor subunit by the binding of biotin-(AC_5_)_2_-hydrazide with the long spacer tail as we expected, we did not have the direct evidence at the present stage. We will reconsider this mechanism for the fluorescence enhancement by the site-directed mutation of Trp79 and Trp108 in the near future.

**Figure 4:**
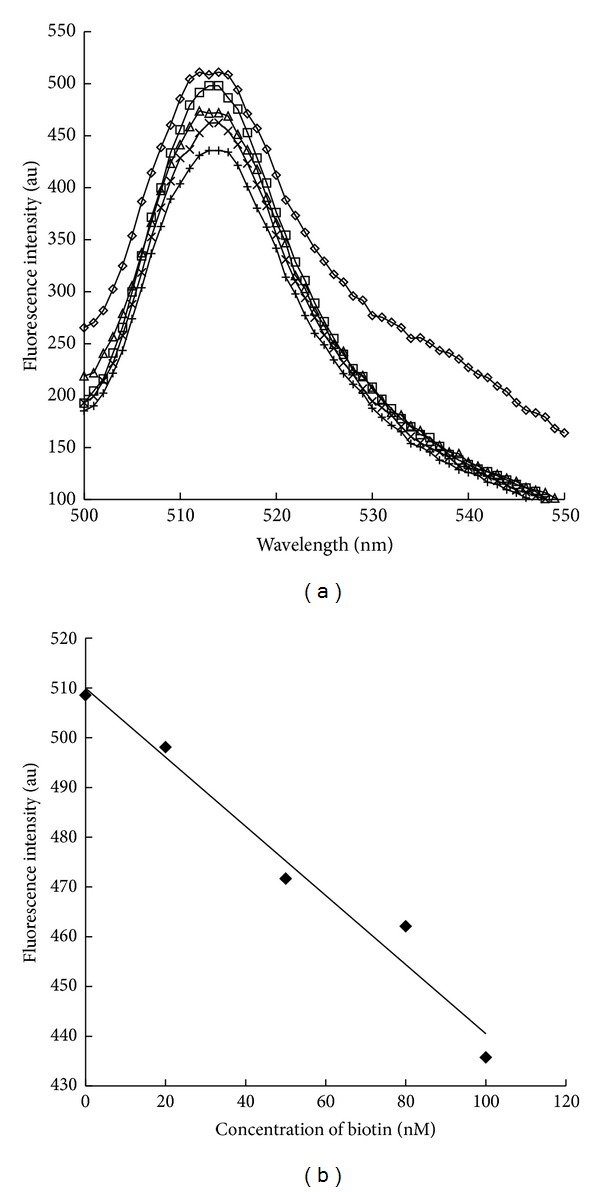
(a) Fluorescence spectra of the Trp120BFLAF mutant streptavidin in the presence of the pairs of 0 nM (–*◊*–), 20 nM (–□–), 50 nM (–▵–), 80 nM (–×–), or 100 nM (–+–) of natural biotin and 100 nM of biotin-(AC_5_)_2_-hydrazide. Marked fluorescence enhancement was observed for the Trp120BFLAF mutant streptavidin by addition of biotin-(AC_5_)_2_-hydrazide. Decrease of fluorescence of Trp120BFLAF mutant streptavidin was observed upon competitive biotin addition in the presence of 100 nM of biotin-(AC_5_)_2_-hydrazide. (b) The fluorescence intensities of the Trp120BFLAF mutant streptavidins in the presence of the pairs of various concentrations of natural biotin and 100 nM of biotin-(AC_5_)_2_-hydrazide at 514 nm were plotted. fluorescence intensity (au) of the Trp120BFLAF mutant streptavidin depended on the concentration of free biotin. All of the fluorescence spectra of mutant streptavidins were excited at 490 nm.

Finally, we applied the fluorescence enhancement of the Trp120BFLAF mutant streptavidin by the binding of biotin-(AC_5_)_2_-hydrazide to the molecular biosensing system for free biotin detection. Streptavidin has very high affinity to biotin, and then the streptavidin-biotin binding was almost irreversible. For accurate quantitative analysis of biotin, we determined the biotin concentration with the following two steps: (i) biotin was first added into the Trp120BFLAF mutant streptavidin solution and (ii) overdose of biotin-(AC_5_)_2_-hydrazide was next added for binding to the mutant streptavidin solution. Fluorescence decrease of the Trp120BFLAF mutant streptavidin with biotin-(AC_5_)_2_-hydrazide (100 nM) was observed upon the addition of natural biotin. Furthermore, biotin concentration dependent decrease of fluorescence intensity of the Trp120BFLAF mutant streptavidin with biotin-(AC_5_)_2_-hydrazide (100 nM) upon competitive addition of biotin was observed (Figures 4(a) and 4(b)). These results demonstrated that the fluorescence enhancement by the pair of the fluorescent Trp120BFLAF mutant streptavidin and biotin-(AC_5_)_2_-hydrazide was successfully applied for biotin sensing. Free biotin was determined in the concentration from 20 to 100 nM in this method.

In this study, not only a new molecular biosensor for biotin was developed, but also a rigorous design to make artificial protein biosensor by using four-base codon method was demonstrated. The result suggested that molecular biosensor for small ligand could be successfully designed in the pair of genetically engineered fluorescent mutant binding protein and ligand analogue.
